# Improving Triage Accuracy in Prehospital Emergency Telemedicine: Scoping Review of Machine Learning–Enhanced Approaches

**DOI:** 10.2196/56729

**Published:** 2024-09-11

**Authors:** Daniel Raff, Kurtis Stewart, Michelle Christie Yang, Jessie Shang, Sonya Cressman, Roger Tam, Jessica Wong, Martin C Tammemägi, Kendall Ho

**Affiliations:** 1 Department of Family Practice Faculty of Medicine The University of British Columbia Vancouver, BC Canada; 2 Department of Emergency Medicine Faculty of Medicine The University of British Columbia Vancouver, BC Canada; 3 Faculty of Health Sciences Simon Fraser University Burnaby, BC Canada; 4 School of Biomedical Engineering Faculty of Applied Science The University of British Columbia Vancouver, BC Canada; 5 Faculty of Medicine The University of British Columbia Vancouver, BC Canada; 6 Computer Science Faculty of Science The University of British Columbia Vancouver, BC Canada; 7 Faculty of Applied Health Sciences Brock University St. Catharines, ON Canada

**Keywords:** telemedicine, machine learning, emergency medicine, artificial intelligence, chatbot, triage, scoping review, prehospital

## Abstract

**Background:**

Prehospital telemedicine triage systems combined with machine learning (ML) methods have the potential to improve triage accuracy and safely redirect low-acuity patients from attending the emergency department. However, research in prehospital settings is limited but needed; emergency department overcrowding and adverse patient outcomes are increasingly common.

**Objective:**

In this scoping review, we sought to characterize the existing methods for ML-enhanced telemedicine emergency triage. In order to support future research, we aimed to delineate what data sources, predictors, labels, ML models, and performance metrics were used, and in which telemedicine triage systems these methods were applied.

**Methods:**

A scoping review was conducted, querying multiple databases (MEDLINE, PubMed, Scopus, and IEEE Xplore) through February 24, 2023, to identify potential ML-enhanced methods, and for those eligible, relevant study characteristics were extracted, including prehospital triage setting, types of predictors, ground truth labeling method, ML models used, and performance metrics. Inclusion criteria were restricted to the triage of emergency telemedicine services using ML methods on an undifferentiated (disease nonspecific) population. Only primary research studies in English were considered. Furthermore, only those studies using data collected remotely (as opposed to derived from physical assessments) were included. In order to limit bias, we exclusively included articles identified through our predefined search criteria and had 3 researchers (DR, JS, and KS) independently screen the resulting studies. We conducted a narrative synthesis of findings to establish a knowledge base in this domain and identify potential gaps to be addressed in forthcoming ML-enhanced methods.

**Results:**

A total of 165 unique records were screened for eligibility and 15 were included in the review. Most studies applied ML methods during emergency medical dispatch (7/15, 47%) or used chatbot applications (5/15, 33%). Patient demographics and health status variables were the most common predictors, with a notable absence of social variables. Frequently used ML models included support vector machines and tree-based methods. ML-enhanced models typically outperformed conventional triage algorithms, and we found a wide range of methods used to establish ground truth labels.

**Conclusions:**

This scoping review observed heterogeneity in dataset size, predictors, clinical setting (triage process), and reported performance metrics. Standard structured predictors, including age, sex, and comorbidities, across articles suggest the importance of these inputs; however, there was a notable absence of other potentially useful data, including medications, social variables, and health system exposure. Ground truth labeling practices should be reported in a standard fashion as the true model performance hinges on these labels. This review calls for future work to form a standardized framework, thereby supporting consistent reporting and performance comparisons across ML-enhanced prehospital triage systems.

## Introduction

Surging emergency department (ED) visits lead to overcrowding in the ED setting, contributing to adverse patient outcomes, staffing challenges, and health system constraints [1]. Challenges in maintaining ED capacity are estimated to cost millions in health care expenditures [2]. Various interventions have been proposed to improve ED conditions, and prehospital telemedicine triage systems are among the most promising, having the potential to prioritize patients based on their likelihood of requiring emergency versus community-based care [3], potentially alleviating the influx of low-acuity patients that would otherwise be managed in the high-cost, resource-intensive ED setting [4,5]. Such systems, including emergency medical dispatch, nurse-staffed telephone lines, and symptom checkers (chatbots), share the common goal to triage patients based on the information that is provided at the first contact for an urgent health concern. These prehospital services often do not include physician assessments, instead using either rule-based algorithms or health personnel for patient triage [6]. In telemedicine, defined as the delivery of health care services at a distance [7], the inherent scarcity of objective or physical measures such as vital signs has spurred efforts to improve risk prediction using machine learning (ML) models applied to a wide array of information sources such as free text from patient intake calls and vital symptoms monitoring [6,8].

Machine learning has been recognized as one option for improving the accuracy of prehospital telemedicine triage systems. To date, ML has commonly been applied in areas of precision medicine (ie, prediction of the success of treatment regimens), though it is rapidly expanding into diverse sectors of health care [9]. In the ED setting, ML models show promise in their ability to accurately predict inpatient admissions and sepsis [10,11]. Incorporating contextual information into ML models can improve prediction of prehospital emergency services [5]. However, there is a lack of understanding and evidence-based practices regarding how ML can optimally be implemented in remote prehospital settings as compared with more data-rich, in-person settings such as the ED [5,10,11].

In supervised learning, ML models learn from labeled data that serve as the “ground truth.” Ground truth refers to the nature of the problem that is the target of the ML model; in the context of prehospital triage, ground truth is the “correct” triage outcome of a patient. The exact process for defining ground truth is complex and substantially varies across studies. Unlike clear binary ground truths, such as “alive” or “deceased,” determining the “correct” triage outcome is more complex, potentially involving subsequent chart reviews or nuanced clinician assessments, thus introducing ambiguity.

A review of the predictors, labels, and models used in prehospital triage systems needs to establish an evidence base for efficient ML methods of triaging patients and identification of gaps not included in the existing models. Without a review, it is unclear whether existing methods and models may generalize to certain settings or be free from biases [12]. Thus, we conducted a scoping review to support our understanding of ML applications in remote prehospital settings. Scoping reviews are useful in the research fields of social sciences and health care, especially when a topic has not yet been comprehensively reviewed or exhibits a large, complex, or heterogeneous nature not amenable to a more thorough systematic review [13]. While there have been literature reviews outlining ML methods in emergency settings [14-16], no specific review touches on prehospital telemedicine triage services. Related reviews such as Sánchez-Salmerón et al [15] focused on in-person triage, as opposed to prehospital and remote triage. Through this scoping review, we aim to explore what evidence exists to compare the effectiveness of ML-enabled strategies with conventional triage methods in improving outcomes for patients seeking care through telemedicine services. We also aim to explore (1) what data sources and approaches are used for extracting meaningful predictors and labels, (2) what models and performance metrics are used, and (3) the processes of telemedicine triage systems where ML has been applied.

## Methods

### Search Strategy

Elements of the PRISMA-ScR (Preferred Reporting Items for Systematic reviews and Meta-Analyses extension for Scoping Reviews) protocol [17] and the Population, Intervention, Comparison, and Outcome (PICO) framework [18,19] were used to guide our search strategy. The search included all articles published prior to February 24, 2023. Methodological frameworks for scoping reviews from Arksey and O’Malley [20] and Levac et al [21] were followed.

The search strategy began with initial searches conducted through MEDLINE to extract terms based on article titles and abstracts. Keywords and expressions included both regular and Medical Subject Headings (MeSH) terms. The following search expression was developed and applied: “Telephone/ or telephone.mp. OR phone.mp. OR telemedicine.mp. OR Telemedicine/ OR Emergency Medical Service Communication Systems/ OR Emergency Medical Dispatch/ OR dispatch.mp. OR hotline.mp. OR Hotlines/ OR prehospital.mp. OR pre-hospital.mp. OR remote consultation.mp. OR Remote Consultation/” AND “machine learning.mp. OR Artificial Intelligence/ OR Machine Learning/ OR artificial intelligence.mp. OR natural language processing.mp. OR Natural Language Processing/ OR chatbot.mp.” AND “triage/ OR triage.mp”

Our search followed the 3 phases listed from the PRISMA-ScR protocol: identification, screening, and inclusion [17] (Multimedia Appendix 1). Using the search expression, articles were retrieved from the following databases: MEDLINE, PubMed, Scopus, and IEEE Xplore. These databases were selected for this review due to their ability to cover the most scientific information in fields such as telemedicine and health care decision-making while also being previously used by several health and technology-related reviews [15,16,22]. In order to limit bias and ensure reproducibility, we elected to exclusively include articles identified through the predefined search strategy.

### Article Selection

All identified records were combined (n=296) and duplicates (n=131) removed resulting in 165 records remaining. The results of the initial search after duplicate removal are found in Multimedia Appendix 2. Articles needed to meet a set of inclusion and exclusion criteria (Table 1), which were developed to ensure that articles were relevant to prehospital telemedicine triage, conducted remotely as opposed to in the ED. During an initial piloting phase, 3 researchers (DR, KS, and MY) piloted these criteria to ensure consistency across them. Each researcher independently screened each title based on the inclusion and exclusion criteria, with discrepancies reaching consensus through discussion, which resulted in 32 records remaining (n=133 excluded). Articles that passed the title screening were then screened for relevant abstracts following a similar process resulting in 15 records remaining (n=17 excluded). The remaining articles were then read in their entirety and all 15 records were deemed eligible and included in the results of the review. Of the articles excluded, most were excluded for the provision of in-person care (as opposed to data collected remotely), were not primary research studies, or were not for general purpose triage (eg, stroke-specific triage).

**Table 1:** Inclusion and exclusion criteria of screening strategy.

PICO^a^/other element	Inclusion criteria	Exclusion criteria
Population	Undifferentiated population seeking emergency services (including COVID-19 assessments).	Specialty-specific population (eg, stroke, heart disease).
Concept	Triage of emergency telemedicine services enhanced by any ML^b^ method that includes only data collected remotely.	Only conventional triage methods used or ML models that include predictors derived from physical assessments (eg, vital signs). Internet of medical things devices requiring physical or in-person assessments were excluded (eg, home blood pressure equipment).
Context	Provision of emergency telemedicine services, including web-based symptom checkers, clinician-staffed telephone line, or emergency medical dispatch.	Provision of in-person emergency care.
Evidence type	Primary research studies.	Literature reviews, protocols, guidelines, letters, gray literature, and qualitative studies excluded.
Language	Studies published in English.	Studies in languages beyond English.
Date	Published before February 24, 2023.	Published on or after February 24, 2023.

^a^PICO: Population, Intervention, Comparison, and Outcome.

^b^ML: machine learning.

### Data Extraction

We extracted data from the included articles with an aim to understand how research in this domain is conducted. A data extraction tool was developed using the JBI Manual for Evidence Synthesis template [23] with a pilot step on 2 sources conducted by 2 researchers (JS and DR). The data of interest fell in four main categories:

1. Study characteristics: Author, year of publication, country of origin, the aim of the study, the result of the study, population assessed, dataset source, dataset size (the number of patient records), prospective/retrospective/deployed, and triage process.
2. ML model predictors and labels: Number of predictors and data types; methods for determining ground truth labels.
3. ML techniques and corresponding performance metrics:
4. Comparators, dataset partitions, data-preprocessing methods, performance metrics and values, and data quality analysis approaches.
5. Resources for future ML model development: Source code availability and software packages used.

This list of extraction items was supported by prior literature that involved emergency care–related triage [22,24] and was refined based on discussions among the authors. For each of the eligible articles included in this review, 1 author (JS) extracted and tabulated the relevant information, and the information was validated by another author (DR) with discrepancies further assessed by a last author (KS). All extracted information was analyzed by the authors to derive a narrative synthesis of the findings. Aligned with the scoping review methodology, articles were not assessed for quality or risk of bias and no statistical analyses were conducted. The data extraction tool and raw data extracted are available in Multimedia Appendix 2.

### Triage Process

We found that the studies spanned 3 prehospital triage processes: emergency dispatch, telephone lines, and chatbot applications. When distinguishing between emergency dispatch and telephone line systems, context of the call and nature of interaction were the 2 main points of consideration. Emergency dispatch calls (eg, 9-1-1) are received and handled by trained emergency staff focused on collecting critical information on the emergency, such as the nature of the incident, location, and immediate risks. The dispatcher then makes decisions based on this information to allocate appropriate resources, such as ambulances or first responders. Conversely, telephone lines such as nurse-led helplines or crisis hotlines are designed to offer support, advice, and clinical guidance to individuals seeking health care information or experiencing a crisis [25]. The interaction is often more conversational and supportive, resulting in a more complex triage process that depends on the nature of the call, the expertise of the health care professionals involved, and the available resources or referrals.

### Predictors

We classified the predictor variables into 4 domains (demographic factors, operational characteristics, clinical factors, and unstructured data such as free text) and extracted how these data were handled in the models. These domains were determined based on the prehospital triage field and iteratively refined as data were extracted.

### Ground Truth Labels

Variability existed in how ground truth labeling methods were coded for ML processing due to diverse sources of training data and annotation methods. In our classification of the observed ground truth methods, we first identified whether the label was derived from (1) the usual clinical process, that is, subsequent clinician triage, or (2) outside of the usual clinical process, that is, post hoc. Within category 1, we further identified whether (1A) remote-only data were used, or (1B) physical, in-person data were used. Within category 2, we further classified whether (2A) labels were automatically derived or (2B) labels were manually derived. Automatically (systematically) derived labels are the results of a uniform and scalable application of a label-deriving algorithm across a dataset.

## Results

### Characteristics of the Studies

**Figure 1:**
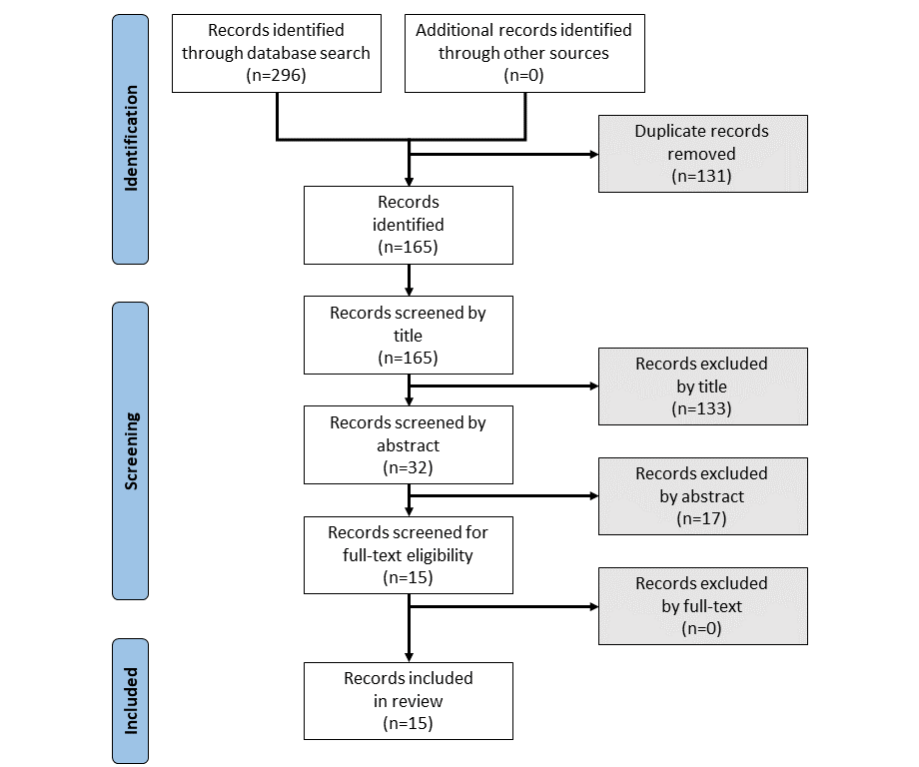
PRISMA-ScR (Preferred Reporting Items for Systematic Reviews and Meta-Analyses Extension for Scoping Reviews) flow diagram.

Figure 1 shows a summary of the search protocol phases followed for this review using the PRISMA-ScR flow diagram. Of the 15 unique studies included, 33% (5/15) of the articles were published in 2022, 26% (4/15) in 2021, 20% (3/15) in 2020, and the remaining 20% (3/15) of the articles between 2014 and 2019. The most frequently occurring country of publication was the United States (4/15, 27%), followed by Japan (2/15, 13%), with the remaining being published in 9 distinct countries (Table 2).

Most studies (11/15, 73%) were retrospective, using historical patient outcomes to assess the performance of ML models in triage prediction. Among the retrospective articles, 2 reported a combination of retrospective results and the performance of deployed models. Of the other 4 nonretrospective articles, 13% (2/15) focused solely on deployed models and 13% (2/15) carried out a prospective study to explore the applications of ML triage in a specific health care setting.

Moreover, 47% (7/15) of articles investigated the use of ML in emergency dispatch calls, 33% (5/15) of articles focused on chatbot-style applications that could be accessed via the internet or within the ED, and 20% (3/15) of articles investigated ML in telephone lines, including 13% (2/15) nurse-led phone lines and 7% (1/15) crisis hotline.

**Table 2:** List of 15 studies included in the review and their characteristics.

Author (year)	Study setting (country)	Dataset size^a^	Labels included	Aim	Triage phase
Anthony et al (2021) [26]	South Africa	93	3 binary	Classify critical conditions in emergency calls	Dispatch
Ceklic et al (2022) [27]	Australia	11,971	1 binary	Classify severity of traffic crash incidents in dispatch calls	Dispatch
Chin et al (2022) [28]	Taiwan	114	1 binary	Classify severity of traffic crash incidents in dispatch calls	Dispatch
Cotte et al (2022) [29]	Germany	385	1 multiclass	Classify triage decisions using a symptom assessment app	Chatbot
Ferri et al (2021) [30]	Spain	1,244,624	3 binary and multiclass	Classify emergency incidents in dispatch calls	Dispatch
Gatto et al (2022) [31]	United States	574	1 binary	Classify severity in patient’s text-based inquiries	Chatbot
Inokuchi et al (2022) [32]	Japan	15,442	1 binary	Identify undertriage in prehospital telephone triage	Nurse-led phone line
Lai et al (2020) [33]	United States	—^b^	—	Classify triage for prehospital COVID-19 cases	Chatbot
Marchiori et al (2021) [25]	Switzerland	>900,000^c^	1 multiclass	Evaluate AI^d^-powered chatbot for symptom-checker triage	Chatbot
Morse et al (2020) [34]	United States	26,646	—	Evaluate AI-powered chatbot for symptom-checker triage	Chatbot
Pacula et al (2014) [35]	United States	427	2 multiclass	Classify triage and distress indicators in crisis hotline chats	Crisis hotline
Spangler et al (2019) [3]	Sweden	68,668	1 continuous	Validate ML^e^-generated risk scores for prehospital care	Dispatch (operated by nurses)
Tollinton et al (2020) [5]	United Kingdom	1,188,509	1 binary	Classify triage of unconscious patients in dispatch calls	Dispatch
Veladas et al (2021) [36]	Portugal	269,669	1 multiclass	Classify clinical pathways from text data	Nurse-led phone line
Yunoki et al (2014) [4]	Japan	61,927	1 multiclass	Classify triage categories from phone call data	Dispatch

^a^Number of patient records included.

^b^Dataset size used for model development was not stated for this study or information on labels was not included.

^c^The study stated that “more than 900,000 case records” were included.

^d^AI: artificial intelligence.

^e^ML: machine learning.

The size of the dataset used across studies varied considerably, with a median sample size of 21,044 observations. The largest dataset comprised 1,224,624 anonymized patient records [30] and the smallest dataset included 93 call transcripts [28]. Distinct methods were reported to handle missing data; for example, Ferri et al [30] excluded all call records with missing values, while Inokuchi et al [32] performed imputation to account for missing data (3918/19,114, 20.5% of cases had missing data) using the k-nearest neighbors (k-NN) algorithm.

### Predictors

**Figure 2:**
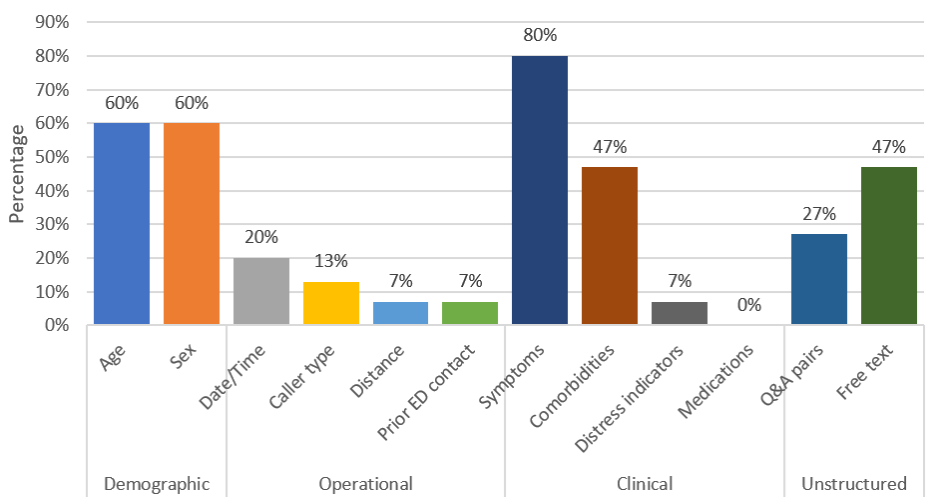
Percentage of predictor types included in the studies. ED: emergency department; Q&A: question and answer.

Several articles used predictors that spanned multiple categories, as exemplified by Ferri et al [30], who extracted patient demographic data (age, gender), operational data (date, caller type), and unstructured data (clinical free-text observations). In contrast, Chin et al [28] used unstructured data (dispatch call transcript) as the only data source in their ML model development, citing the “higher expressiveness of patient condition than structured data.” The frequency of occurrence for each of these domains is shown in Figure 2.

The mapping and processing techniques varied across data types. Most commonly, many input variables were one-hot encoded, such as age groupings, sex, day of week, clinical indicators (symptoms, comorbidities), and question-answer pairs. Notably, distance to ED was one of the few continuous predictors used [3]. For unstructured data, various natural language processing (NLP) techniques were employed to transform these data to useful inputs, such as bag-of-words methods or text vectorization, which are strategies to transform text data into a numerical representation that can be processed by ML models (see Multimedia Appendix 3 for more information).

### Data Labeling: Determining Ground Truth

**Figure 3:**
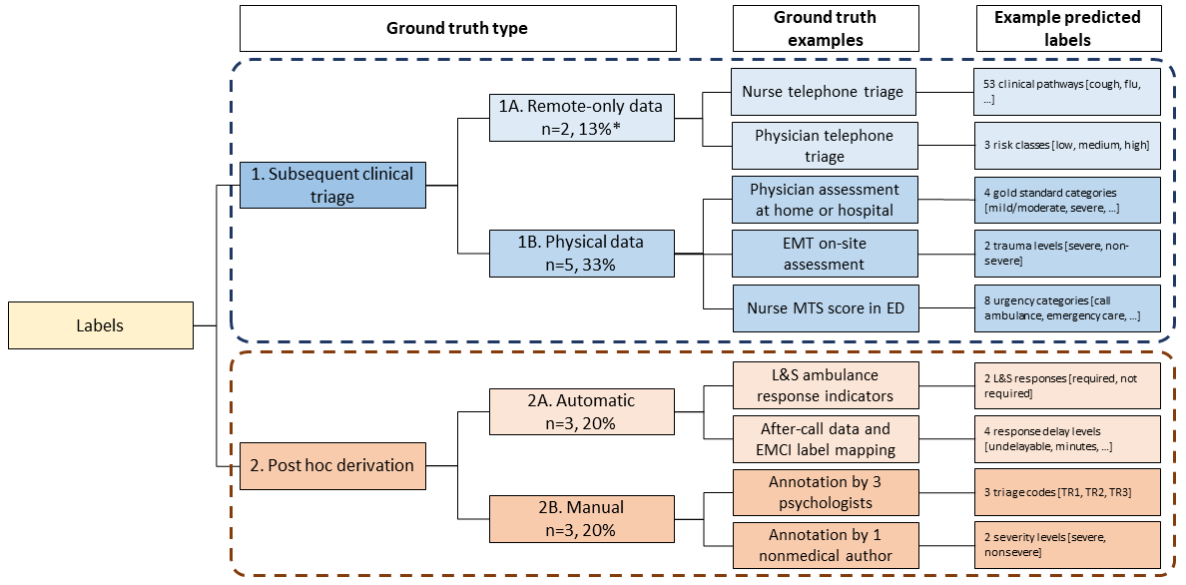
Label and ground truth methodologies by annotation method. *Marchiori et al used remote physician triage as ground truth for model training but manually derived labels for model testing. To ensure consistency in article classification, we have categorized all articles based on the ground truth method for model training. ED: emergency department; EMCI: emergency medical call incident; EMT: emergency medical technician; L&S: lights and sirens; MTS: Manchester Triage System.

Two studies (2/15, 13%) [33,34] did not provide sufficient information to determine their ground truth labeling method. Most of the ground truth methods (5/15, 33%) used physical assessments from the usual clinical process; however, within this group input data were from 3 different types of providers (nurses, physicians, and ambulance crew) with each employing a different triage or categorization system (Figure 3) [25]. This results in variability as similar information is outputted with different labels, which limits comparability (eg, patient conveyance [5] or “gold standard” ED triage protocols [4]). Notably, the 3 (20%) studies [3,27,30] that used automatic post hoc (2A) methods had larger sample sizes (mean 4,32,792 observations; range 15,550-1,244,624) versus studies [26,31,35] using manual (2B) methods (mean 364 observations; range 93-573). Across the 4 methods, 6 studies (40%) predicted labels that were binary and based on symptom or condition severity (eg, life-threatening [30], severe trauma [28]), while 7 studies (47%) predicted multiclass labels (eg, 1 of 53 clinical pathways [36]).

### Interrater Reliability

Methods to enhance interrater reliability were rarely employed, in contrast to published guidelines concerning manual data labeling [37]. Marchiori et al [25] did so by ensuring the adequate training of data collectors by selecting “only records generated by top-ranked doctors (based on years of experience and internal audits).” In the study by Gatto et al [31], data annotators “invested significant time to educate themselves on the symptoms” observed in the dataset; however, it was noted “that the use of nonmedical professionals limited the degree of granularity with which the dataset was labelled.” Pacula et al [35] used consensus among 3 evaluators when labeling data and measured the interannotator agreement using the Fleiss kappa statistic to confirm good agreement.

### ML Algorithms

Most studies applied more than 1 ML technique to method development (11/15, 73%). However, 2 studies (13%) did not disclose the ML algorithm used in their triage prediction model. Three (20%) studies published the source code for various stages of their ML model development and validation (Table 3).

Overall, the most popular ML algorithm was support vector machine (SVM; 8/15, 53%) and tree-based methods, including random forest (RF; 6/15, 40%) and extreme gradient boosting (XGBoost; 4/15, 27%). Other popular algorithms were neural networks (NN; 5/15, 33%), with various implementations observed, such as deep NNs, bidirectional long short-term memory models, and ensemble of deep learning networks (ie, the model employed by Ferri et al [30] is described as a “Deep Ensemble Multitask Classifier for Emergency Medical Calls” composed of 4 different subnetworks). Naïve Bayes (NB) and k-NN were similarly popular, followed by regression methods.

**Table 3:** Type and frequency of ML algorithms used by the included studies.

ML^a^ algorithm	Studies, n	Authors (citations)
Support vector machine	8	Anthony et al [26], Ceklic et al [27], Chin et al [28], Gatto et al [31], Inokuchi et al [32], Pacula et al [35], Spangler et al [3]^b^, and Veladas et al [36]
Random forest	6	Anthony et al [26], Ferri et al [30], Inokuchi et al [32], Tollinton et al [5], Spangler et al [3], and Veladas et al [36]
Neural networks	5	Ceklic et al [27], Ferri et al [30], Gatto et al [31], Inokuchi et al [32], and Marchiori et al [25]
Extreme gradient boosting (XGBoost)	4	Ferri et al [30], Inokuchi et al [32], and Spangler et al [3]
Naïve Bayes	4	Ceklic et al [27], Chin et al [28], Ferri et al [30], and Veladas et al [36]
K-nearest neighbors	4	Anthony et al [26], Ceklic et al [27], Chin et al [28], and Gatto et al [31]
Logistic regression	3	Anthony et al [26], Ferri et al [30], and Spangler et al [3]
Bayesian network	2	Cotte et al [29] and Yunoki et al [4]
Decision tree^c^	2	Chin et al [28] and Tollinton et al [5]
Ensemble^d^	2	Ceklic et al [27] and Ferri et al [30]
LASSO^e^ regression	1	Inokuchi et al [32]
Multilayer perceptron	1	Chin et al [28]
Hidden Markov model	1	Pacula et al [35]
Hierarchical attention network	1	Gatto et al [31]
Transformer-based	1	Gatto et al [31]
Unspecified algorithms	2	Lai et al [33] and Morse et al [34]

^a^ML: machine learning.

^b^Spangler et al [3] did not include support vector machine, random forest, neural networks, or regression models in their “Methods” but stated in their “Discussion” that they investigated these algorithms.

^c^Decision trees here include methods such as gradient boosting but not random forest or extreme gradient boosting.

^d^Ceklic et al [27] did not provide information about the specific models incorporated into their ensemble. Ferri et al [30] built their ensemble using a collection of deep learning subnetworks.

^e^LASSO: least absolute shrinkage and selection operator.

### Model Performance Metrics

*F*_1_-score was the most used metric (6/15, 40%) to measure model performance, closely followed by accuracy (5/15, 33%) and area under the curve (AUC; 4/15, 27%). Furthermore, many studies tangentially used other quantitative measures, such as sensitivity or recall and specificity or precision. In the case where AUC scores were similar between the RF and XGBoost models in the study by Tollinton et al [5], analysis of other metrics showed that XGBoost outperformed RF models in terms of sensitivity (0.93 vs 0.62 in the combined model) but had lower specificity (0.17 vs 0.56 in the combined model). Moreover, several studies investigated how different combinations of predictors and NLP techniques affected model performance across ML techniques. For instance, Pacula et al [35] present an interesting discussion on the impact of various approaches to dialogue processing (turn-level classification and accounting for speaker role) on triage prediction outcomes.

A summary of the comparators and top-performing models for each study, alongside information on evaluation metrics used and data train or test split, is shown in Table 4. For models that were evaluated against non–ML-enhanced methods of triage, those established triage systems are indicated as well.

**Table 4:** Top-performing ML algorithms used in each study and corresponding performance metric and model training or testing split grouped by triage process.

Triage process and study		Top-performing model	Model comparators	Triage comparators	Performance metrics	Training data split (%)
**Emergency medical dispatch**						
	Anthony et al [26]	SVM^a^	LR^b^, RF^c^, k-NN^d^	—^e^	Accuracy	80
	Ceklic et al [27]	Ensemble	k-NN, SVM, NB^f^, deep NN +NLP^g^	SJ-WA^h^ dispatch	PPV^i^, sensitivity (recall), *F*_1_-score	60
	Chin et al [28]	Bernoulli NB	k-NN, DT^j^, SVM, NB +NLP	Dispatcher evaluation	Accuracy, PPV, NPV^k^, sensitivity (recall), specificity	91
	Ferri et al [30]	Ensemble	NB, LR, RF, GB^l^ +NLP	Clinical decision tree	Accuracy, sensitivity (recall), PPV, *F*_1_-score	80
	Spangler et al [3]	XGBoost^m^	SVM, LR, RF, deep NN	Dispatch priority, National Early Warning Scores	AUC^n^, PPV, sensitivity (recall)	66
	Tollinton et al [5]	GB/RF^o^	—	—	AUC, sensitivity (recall), specificity	80
	Yunoki et al [4]	BN^p,q^	—	—	Accuracy	90
**Symptom checker/chartbot^r^**						
	Cotte et al [29]	BN^q^	—	MTS^s^	Cohen κ	N/A^t^
	Gatto et al [31]	SBERT^u^	BERT^v^, SVM, HAN^w^, bi-LSTM^x^ +NLP	—	PPV, sensitivity (recall), *F*_1_-score	80
	Marchiori et al [25]	Bi-LSTM	Convolutional NN, recurrent NN +NLP	—	PPV, sensitivity (recall), *F*_1_-score	60
**Telephone line**						
	Inokuchi et al [32]	RF^q^	LASSO^y^ regression, deep NN, XGBoost	—	Area under the receiver operating characteristic curve, PPV, NPV, sensitivity (recall), specificity	70
	Pacula et al [35]	SVM^q^	HMM^z^ +NLP	—	AUC, *F*_1_-score	82
	Veladas et al [36]	SVM	RF, NB +NLP	—	Accuracy, *F*_1_-score, PPV, sensitivity (recall)	64

^a^SVM: support vector machine.

^b^LR: logistic regression.

^c^RF: random forest.

^d^K-NN: K-nearest neighbors.

^e^No comparator.

^f^NB: naïve Bayes.

^g^“+NLP” indicates that model performance was evaluated across various natural language processing techniques.

^h^SJ-WA: St John Ambulance in Western Australia.

^i^PPV: positive predictive value (precision).

^j^DT: decision tree.

^k^NPV: negative predictive value.

^l^GB: gradient boosting.

^m^XGBoost: extreme gradient boosting.

^n^AUC: area under the curve.

^o^The gradient boosting model scored better on sensitivity, but specificity was lower than RF.

^p^BN: Bayesian network.

^q^Alternate ML models were not compared.

^r^Lai et al [33] and Morse et al [34] reported on symptom checker triage systems but did not provide details on the underlying ML model and comparators or development.

^s^MTS: Manchester Triage System.

^t^N/A: not applicable.

^u^SBERT: sentence bidirectional encoder representation from transformers.

^v^BERT: Bidirectional Encoder Representation From Transformers.

^w^HAN: hierarchical attention network.

^x^Bi-LSTM: bidirectional long short-term memory.

^y^LASSO: least absolute shrinkage and selection operator.

^z^HMM: hidden Markov model.

### Model Data Splitting

Thirteen studies (87%) reported some method for validation, including randomly partitioning data into training or testing sets (10/15, 67%) or training or validation or testing sets (3/15, 20%). Most studies used some form of cross-validation in the training sets for model construction (8/15, 53%). In addition, various types of resampling procedures were used, such as *k*-fold cross-validation, stratified *k*-fold cross-validation, repeated *k*-fold cross-validation, or repeated random test-train splits.

## Discussion

### Principal Findings

We reviewed 15 primary research studies of ML-enhanced triage models in prehospital telemedicine settings where patients potentially require emergency care. We found that ML-enhanced triage systems typically outperformed conventional triage ones; however, there is likely a bias toward publishing these types of positive findings [38]. While the reviewed studies exhibited several commonalities in terms of predictor types, ML algorithms tested, and performance metrics, there were key discrepancies in how data were sourced and processed, particularly with regard to annotating ground truth labels. We discuss these similarities and differences in context and how the new evidence presented here relates to ED overcrowding and prehospital triage.

### Predictor Variables

A critical limitation of prehospital telemedicine triage systems is the lack of access to objective measures of the patient condition typically obtained through physical assessment, vital signs being fundamental metrics of illness severity. As a result, our inclusion criteria ensured that no study had access to a physical assessment of their participants. Therefore, by investigating the range of predictors employed by the included articles, we identified how subjective and indirect indicators of patient condition are used in remote triage. Our review indicates that patient symptoms, age, sex, and comorbidities were the most frequently occurring predictor variables among structured data, with NLP techniques used to extract features from unstructured data. The structured and unstructured data were not combined in any of the included articles. This finding emphasizes the critical importance of considering these 4 clinical and demographic factors for prehospital telemedicine triage where physical assessment data are unavailable. The consistent use of patient symptoms, demographics, and comorbidities as predictor variables across all 3 triage processes reinforces that they are reliable indicators of a suitable triage outcome. However, while demographic factors were frequently used across the studies, only 2 specific factors, namely, age and sex, were considered.

Notably, we also highlight the absence of previously identified important inputs to ML models [12]. Race or ethnicity, region or geography, medication history, and health system exposure (hospitalizations, etc) were not represented in the corpus; however, they should be taken into consideration in prehospital triage both to improve performance and ensure algorithmic fairness [39]. The absence of these variables both as predictors and for post hoc evaluation of algorithmic bias suggests a significant gap in the extant literature and motivates further investigation into their potential to generate accurate and equitable triage outcomes for more diverse populations. Continuing to develop a comprehensive list of the most significant variables driving remote emergency triage is invaluable to improving equitable patient outcomes for all populations [12]. Identifying common patterns in predictor selection (and what is absent) can inform the development of standardized guidelines for building ML algorithms for triage using remote-only data. A future with consistent measurements of physiologic metrics, such as vital signs, would also be invaluable to strengthen prediction. Developing systems or technology whereby these critical data can be captured remotely is a future direction worth exploring.

### Ground Truth Labeling Methods

Our review critically spotlights the variety of methods for annotating data labels from ground truth. Two papers did not provide sufficient information, and we classified the remaining 13 into 1 of 4 distinct methods, reflecting varying ML development philosophies. The most common method—(2A) post hoc automatic derivation of data labels—indicates preference for collaboration among domain experts to reduce human subjectivity and implement large-scale data mapping. Notably, Ferri et al [30] used a panel of 17 physicians to develop a mapping system, which was then automatically applied to more than 1 million records. In contrast, using the (2B) method, Pacula et al [35] reported that 3 psychologists manually annotated each of the 427 records. While this review cannot comment on the comparative efficiencies of these specific methods, we note that assembling large datasets with ground truth labels is an arduous and expensive task; thus, there may be scalability benefits to automatic methods [40].

While we observed trade-offs in data labeling, establishing methodologically robust ground truth is of paramount importance. Inadequate representation of ground truth can lead to misclassification issues within the ML model, thus reducing predictive accuracy on external data. This could result in unintended and potentially serious consequences, especially in the context of emergency care. The ground truth triage outcome has proved challenging to pinpoint, and this scoping review reveals 4 methods in determining it. However, each method exhibits limitations and potential manifestations of human subjectivity.

The accuracy of remote triage is inherently limited by the absence of physical data, even when carried out by individuals with domain expertise (eg, physicians). Furthermore, human bias and lack of experience can lead to either overtriage (ie, false positives; eg, sending low-acuity patients to the ED) or undertriage (ie, false negatives; eg, advising high-acuity patients to stay home), even in cases when physical data are available. To address this, the strategy of selecting data from only highly reputable clinicians [25] serves as an inspiration for developing a standardized evaluation system to identify qualified data annotators for health care settings. An additional method to enhance triage accuracy is inclusion of downstream triage with access to more comprehensive data, such as vital signs, detailed physical assessment, or a professional with higher-level training (eg, a physician conducting a home visit) [32].

The methodical classification of patients based on a predetermined list of outcomes minimizes the risk for interrater disagreement. However, relying solely on these selected outcomes, such as hospital admission or 2-day mortality [3], assumes that they are the only factors and of equal importance in determining the overall risks associated with the patient. This approach overlooks insights that can be revealed from a circumstantial and personalized analysis of patient condition. In addition, many of the inclusion studies selected highly specific outcomes, such as sepsis, myocardial infarction, and cardiac arrest [26], which are not generalizable to different remote triage processes. One potential solution, which no study in this review used, is using a weighted kappa index to consider different categories and disagreements and capture the rank magnitude of disagreement [41].

Retrospective evaluation of patient data by multiple annotators holds potential for highly accurate ground truth labels, as it is the most comprehensive approach observed in this review. However, we note that the included articles lack information regarding how evaluators determined such classifications. Subjectivity in human decision-making persists and variation in annotators’ levels of training and resolution strategies employed leaves room for further research. Again, implementing a standardized evaluation system to determine a qualified pool of data annotators becomes crucial to ensure reliability in the annotation of ground truth triage labels for ML-enhanced remote triage [37].

This analysis of subjectivity in ML systems underscores the need for nuance regarding objectivity and ground truth. To enhance remote triage algorithm generalizability, comprehensive datasets must be used. This entails capturing data from each of the predictor variables outlined in this review, as well as race or ethnicity, region or geography, and medical history. Furthermore, standardized protocols for data labeling across different clinical settings would facilitate consistency and enable meaningful comparisons and analyses. There is a need for more well-defined and externally valid procedures for determining the ground truth in the context of highly intricate and unstructured data such as remote patient triage.

### ML Techniques

This review presents needed insights on the cutting-edge application of ML methods for remote prehospital triage systems: SVM, tree-based, and NN methods were commonly used. These observations are consistent with other reviews of ED-based implementations [16,42]. To our knowledge, this is the first review to provide a mapping between ML model development and 3 processes of triage in emergency medicine: emergency medical dispatch, chatbot or symptom checker, and telephone line. We uniquely charted the best-performing model of each article to 1 of the 3 triage processes and generated insight on the specific elements underpinning each model. By doing so, we provide insight into how predictive ML models can be developed for different stages of triage before physical assessment.

### Limitations

We did not directly compare the varied performance metrics such as the *F*_1_-score, precision, recall, and AUC, as this could lead to misleading conclusions. This was not an objective of our review due to inconsistencies in reported metrics and variability in study designs. For instance, certain metrics can be optimized at the expense of other metrics; for example, precision and recall are inversely related. Not all metrics are universally applicable; the *F*_1_-score is unsuitable for multiclass problems found in a proportion of included studies. In addition, *F*_1_-score assumes false negatives and false positives are equally costly, an incorrect assumption in triage problems where undertriage due to a false negative would have serious consequences. While AUC is perhaps the optimal metric choice, it still has sensitivity to class imbalances, which varied across the included studies. Of the only 4 articles that reported AUC, the performance ranged from 0.64 for prediction of conveyance [5] to 0.88 [35]. A direct comparison among articles reporting AUC was avoided due to differences in study contexts, which included diverse triage labels, significant variations in sample sizes and populations, limited reporting on class imbalance, and the use of different validation strategies. As this review did not facilitate any quantitative analysis of ML model performance, insights into the accuracy of ML-enhanced triage compared with conventional remote triage, as well as the quantitative impact that ML triage may have on patient outcomes and overall health care systems, could not be derived and future systematic work is warranted.

### Implications for Future Work

ML-enhanced triage presents an opportunity to alleviate the burden on EDs and support patients’ decision-making when seeking emergency versus community-based care. Based on the evidence synthesized here, our calls to the field are to determine a prioritized list of high-value predictor variables to consider, standardize ground truth labeling, and form a consensus on validation methods used, such that different health systems can continuously learn from new developments. This review provides a foundation for developing guidelines, which will also create opportunities for comparison across studies to quantitatively assess the effectiveness and benefits of ML-enhanced triage regarding patient outcomes and health system performance. While reducing resources spent on overtriage is also a priority, it is equally important to focus on the likelihood of undertriage, as it poses a significant risk to patients. Therefore, precision, recall, and specificity rates, as well as algorithmic bias [39], must be carefully monitored and improved in model development to ensure that safety and effectiveness are balanced in ML-enhanced triage systems.

Accurate triage recommendations do not guarantee that patients will follow them [43-45]. In the discussion of using telephone lines or chatbots during the triage process, further investigation is warranted into how delivery methods affect patients’ likeliness to adhere to the advice generated by ML-enhanced triage systems. Similarly, in the dispatch and telephone line triage processes, a question that arises is whether ML-generated triage results influence the decision-making process of dispatchers or providers. This area of research could provide insight on the most effective stage at which ML-based assistance should be introduced in the triage process. Exploring the interplay between ML-enhanced triage advice, patient behavior, and clinician decision-making will contribute to the optimization of prehospital telemedicine triage in emergency care.

### Conclusions

Our scoping review of 15 recent studies of ML-enhanced prehospital telemedicine triage systems observed heterogeneity in dataset size, predictors, clinical setting (triage process), and reported performance metrics. Consequently, a comparison of ML performance across articles was not feasible, and we note that identifying the most efficient and accurate ML-enhanced triage system is valuable for future development and model deployment in prehospital settings, where a standardized performance metric such as the AUC would be important to facilitate comparisons. Standard structured predictors, including symptoms, age, sex, and comorbidities, across articles suggest the importance of these inputs; however, there was a notable absence of other potentially useful data, including medications and health system exposure. With advancing technology of transformer-based models [46,47], there exists the potential for combining structured and unstructured data; an approach that was also absent in the included articles. The lack of social variables leaves the potential for algorithmic bias critically unexplored. Ground truth labeling practices should be reported in a standard fashion as the true model performance hinges on these labels. This review establishes an evidence base for future investigations and an opportunity to form a consensus and standardized framework, thereby supporting consistent reporting, performance comparisons, and collaboratively developed ML-enhanced prehospital triage systems.

## Appendix

Multimedia Appendix 1 PRISMA-ScR (Preferred Reporting Items for Systematic reviews and Meta-Analyses Extension for Scoping Reviews) checklist.

Multimedia Appendix 2 Data extraction tool and search results.

Multimedia Appendix 3 Additional findings.

## Abbreviations

AUC: area under the curve
ED: emergency department
K-NN: K-nearest neighbors
ML: machine learning
NB: naïve Bayes
NLP: natural language processing
NN: neural network
RF: random forest
SVM: support vector machine
XGBoost: extreme gradient boosting
