# Supplementary material for: Improving Triage Accuracy in Prehospital Emergency Telemedicine: Scoping Review of Machine Learning–Enhanced Approaches
Source: Interact J Med Res. 2024 Sep 11;13:e56729. doi: 10.2196/56729 (PMC11429666; doi:10.2196/56729)
Supplement: Multimedia Appendix 2 [file ijmr_v13i1e56729_app2.zip › IJMR_data_extraction_tool.docx]

Data Extraction Tool

1. Author
2. Year of publication
3. Country of origin
4. Aim of study
5. Result of study
6. Population assessed - include age, years of records, source, health system, and other details
7. Prospective/Retrospective/Deployed
8. Triage agent (eg: Nurse line, EMS, chatbot, etc)
   1. Triage phases - an abstraction of triage agent to map to broad categories - nurse-led phone line, dispatch, chatbot, or other categories
9. Triage clinical flow (eg: after chatbot -> nurse; after nurse-line -> physician)
10. Machine learning methods included?
11. Dataset source
12. Dataset size
13. Dataset partitions (training, validation, etc)
14. Predictors
    1. Number of predictors - number of predictors (if different for different models include best performing model and range for others)
    2. (data types - tabular, voice, unstructured text, or combination)
15. Labels
    1. Including how ground truth was derived:
       1. Either systematically or manually
       2. Either with subsequent physical (in-person) assessments or not
16. Comparators
17. *If data processing, what methods used?* (eg: if NLP for unstructured text what models, if voice analysis, what models)
18. Ensure predictors exclude physical/in-person data
19. Type of ML model(s) used
20. If deep learning - data augmentation, regularization methods, etc.
21. Performance metrics and values
22. Data quality analysis - ex: was concept drift addressed? Inter-rater reliability? Other data quality issues?
23. Source Code Availability (T/F)
24. Packages used
25. AUC/AUROC if reported
