# Supplementary material for: Improving Triage Accuracy in Prehospital Emergency Telemedicine: Scoping Review of Machine Learning–Enhanced Approaches
Source: Interact J Med Res. 2024 Sep 11;13:e56729. doi: 10.2196/56729 (PMC11429666; doi:10.2196/56729)
Supplement: Multimedia Appendix 3 [file ijmr_v13i1e56729_app3.docx]

**Predictor Variables**

Demographics data: Age, a discrete variable measured in years, was commonly mapped to intervals (<19, 20-29, 30-39...) and one-hot encoded. One-hot encoding produces a vector of length equal to the number of categories present, with the digit “1” indicating the category that a data point belongs to. Uniquely, age was mapped to a fuzzy representation through piecewise linear functions in Ferri et al. [30]. Sex, a structured and categorical variable, was consistently one-hot encoded.

Operational data: Month and hour of call - discrete variables - recorded in Spangler et al. [3], and whether the call was received on a weekend - a categorical variable - recorded in Ferri et al. [30], were all one-hot encoded. Other operational variables including caller type and prior contact points were also subject to one hot encoding across the articles. Notably, within the 15 articles, Haversine distance to ED recorded in Spangler et al. [3] the single continuous predictor variable.

Clinical data: Multi-categorical variables including symptoms, distress indicators, and comorbidities were also one-hot encoded across the articles. Additional measures were taken for sequential clinical questions, for example, in Spangler et al. [3], questions with multiple potential answers were mapped using ordinal encoding.

Unstructured data: Various natural language processing (NLP) pipelines are employed across the articles to enumerate unstructured data, including the TF-IDF algorithm, bag-of-words, and text vectorization. Additionally, the complexities "speaker vs. chatter" roles in dialogue classification are discussed in Pacula et al. [35].

**Ground Truth and Data Labels**

Ground truth data labels in category 1 are purely observational data - referring to the actual triage outcome determined within the clinical process. In the inclusion corpus, two (15%) articles considered remote (1A) patient assessment facilitated by registered nurses and physicians to be ground truth.

Three articles (23%) considered in-person (1B) patient assessment to be ground truth. Yunoki et al. [4], considered “categories determined by physicians after arrival at a hospital (gold standard) to be the correct responses.” Similarly, Inokuchi et al. [32] utilized patient classification by a physician during a home visit as ground truth, and in Cotte et al. [29], the Manchester Triage System (MTS) score given by a triage nurse in the ED was treated as ground truth.

Category 2 identifies ground truth labels which were derived outside of the clinical process, based on retrospective analysis of patient data. Of the inclusion articles, six (40%) articles developed a procedure to automatically label the data (2A). For instance, Ceklic et al. [27] assigned a label of requiring a “lights and sirens” (L&S) response if the patient had experienced any one of the following: mortality before hospital admission, received L&S transport to hospital, or had one or more high-acuity indicators” from a pre-determined list of critical interventions, observations, and medications. These parameters in which triage categorization were derived from varied drastically across the articles. In Anthony et al. [26], data labelling was based on “true data of patient time-critical conditions; sepsis, myocardial infarction and cardiac arrest.” In the remaining two (15%) articles, evaluators manually analyzed patient data to derive ground truth triage labels (2B). Each patient record was annotated by three authors in Gatto et al. [31], and three psychologists in Pacula et al. [35].
